# Bioaerosol concentrations generated from toilet flushing in a hospital-based patient care setting

**DOI:** 10.1186/s13756-018-0301-9

**Published:** 2018-01-26

**Authors:** Samantha D. Knowlton, Corey L. Boles, Eli N. Perencevich, Daniel J. Diekema, Matthew W. Nonnenmann, CDC Epicenters Program

**Affiliations:** 1 Department of Occupational and Environmental Health, College of Public Health, University of Iowa, 100 CPHB, S346 CPHB, Iowa City, IA 52242 USA; 2 University of Iowa Health Care, Iowa City, IA 52242 USA; 3 Department of Epidemiology, College of Public Health, University of Iowa, Iowa City, IA 52242 USA

**Keywords:** Health care, Infection, Aerosol, Toilet, Flushing, Exposure, Bioaerosol, Bacteria

## Abstract

**Background:**

In the United States, 1.7 million immunocompromised patients contract a healthcare-associated infection, annually. These infections increase morbidity, mortality and costs of care. A relatively unexplored route of transmission is the generation of bioaerosols during patient care. Transmission of pathogenic microorganisms may result from inhalation or surface contamination of bioaerosols. The toilet flushing of patient fecal waste may be a source of bioaerosols. To date, no study has investigated bioaerosol concentrations from flushing fecal wastes during patient care.

**Methods:**

Particle and bioaerosol concentrations were measured in hospital bathrooms across three sampling conditions; no waste no flush, no waste with flush, and fecal waste with flush. Particle and bioaerosol concentrations were measured with a particle counter bioaerosol sampler both before after a toilet flushing event at distances of 0.15, 0.5, and 1 m from the toilet for 5, 10, 15 min.

**Results:**

Particle concentrations measured before and after the flush were found to be significantly different (0.3–10 μm). Bioaerosol concentrations when flushing fecal waste were found to be significantly greater than background concentrations (*p*-value = 0.005). However, the bioaerosol concentrations were not different across time (*p*-value = 0.977) or distance (*p*-value = 0.911) from the toilet, suggesting that aerosols generated may remain for longer than 30 min post flush. Toilets produce aerosol particles when flushed, with the majority of the particles being 0.3 μm in diameter. The particles aerosolized include microorganisms remaining from previous use or from fecal wastes. Differences in bioaerosol concentrations across conditions also suggest that toilet flushing is a source of bioaerosols that may result in transmission of pathogenic microorganisms.

**Conclusions:**

This study is the first to quantify particles and bioaerosols produced from flushing a hospital toilet during routine patient care. Future studies are needed targeting pathogens associated with gastrointestinal illness and evaluating aerosol exposure reduction interventions.

## Background

In the United States (US), 1.7 million people contract an infectious disease from a hospital-based patient care setting [i.e.*,* healthcare associated infection (HAI)], annually [1]. These HAIs increase morbidity, mortality with costs estimates from 4.5 to 29 billion US dollars, annually and are underreported [2, 3].

Infections are easily transmitted in healthcare settings due to a large proportion of sick and immunocompromised individuals [4]. Healthcare staff may unknowingly transmit diseases among patients through patient handling and from contact with contaminated surfaces [5–9]. Hand washing and environmental cleaning are effective methods to remove infectious microorganisms, however not all environmental surfaces are cleaned appropriately [10]. The isolation of microorganisms from hospital surfaces after contamination suggests that infections occur among individuals after being admitted [11–14].

Surface contamination may also occur from bioaerosols generated from infected patients or contaminated equipment. Studies examining healthcare settings have measured bioaerosol concentrations and characterized their composition to understand the generation source of bioaerosols during patient care [15–21]. The bioaerosols measured were composed primarily of bacteria identified as normal flora or infectious organisms [15–21]. Some of the identified organisms cause gastrointestinal illness, raising the concern that toilets are aerosolizing fecal waste [15, 17, 22–24].

Toilet flushing aerosolizes fecal waste from the movement of toilet water (i.e., bubbling, swirling, splashing) during a flushing event [25]. Hutchinson (1956) isolated bacterial species found in fecal matter from toilets and bathroom surfaces. To determine how those surfaces are contaminated, several studies have aerosolized bacteria seeded in toilets. The target bacteria were detected in the air suggesting toilets are generators of bioaerosols [22, 23, 26–28]. Newsom [23] also used normal and homogenized wastes to seed toilets. Higher bioaerosol concentrations were measured from flushing homogenized wastes suggesting loose fecal wastes may result in higher concentrations.

After flushing, residual microorganisms may exist on toilet walls to be later aerosolized [22, 26]. Darlow and Bale [22] and Gerba et al., [26] found a 99% reduction of microorganisms after the first flush but little reduction with subsequent flushes. Barker and Jones [29] and Best et al., [27] seeded toilet walls to mimic splashing of loose fecal wastes when using the toilet. Both studies isolated the microorganisms in the air to suggest bacterial agents in loose fecal wastes can be aerosolized to cause environmental contamination and occupational exposures [27, 29].

Bioaerosols produced from flushing loose fecal wastes excreted from individuals in a healthcare setting have not been investigated. The lack of information is surprising given the rate of HAIs and evidence of bioaerosol generation during flushing. Logistical challenges of sampling flushing in a patient care setting may be a barrier. In addition, the lack of bioaerosol exposure standards may lead to challenges with interpretation. The lack of information is concerning as immunocompromised patients may be affected with relatively small exposures. Furthermore, understanding the production of bioaerosols from flushing fecal waste during patient care is important for determining sources of environmental contamination and developing controls to reduce worker exposure and HAIs.

Prior studies of bioaerosol generation during toilet flushing have not been performed during routine patient care in hospital settings. Furthermore, previous studies did not investigate background concentrations of bioaerosols, particle concentrations, or compared results to flushing of toilets with and without unmanipulated loose fecal wastes. Therefore, the experiments performed in our study were to:

1. Compare the particle concentrations measured before and after a toilet flush across various particle diameters using an aerosol particle counter.
2. Compare the bioaerosol concentrations of three experimental bathroom conditions: no waste no flush, no waste with flush, and waste with flush with bioaerosol impactors.
3. Compare the effect of time and distance on the bioaerosol concentrations across experimental conditions.

## Methods

### Experimental conditions

A hospital-based patient care setting was used for this experiment. The hospital has an 811-bed capacity that annually admits more than 36,000 patients for in-patient hospital care. Three bathroom conditions were evaluated in this hospital. Toilets within these bathrooms were equipped with toilet seats (Kohler Inc., USA), vacuum breakers (V-500-AA/V-600-AA; Sloan Valve Company; USA), and toilet base (Kohler Inc. USA, Crane Inc., USA) which operated at a flush volume of 6.1 L. Background concentrations of bioaerosols and particles were determined from bathrooms where toilets were not flushed and designated as “no waste no flush.” The second condition of “no waste with flush” was to determine if particle and bioaerosol concentrations were produced from residual microorganisms from previous toilet use. The third experimental condition, “fecal waste with flush,” was to determine if particle and bioaerosol concentrations could be produced from flushing loose fecal wastes excreted by patients in a hospital-based patient care setting.

### Air sampling apparatus used in bathrooms

**Fig. 1:**
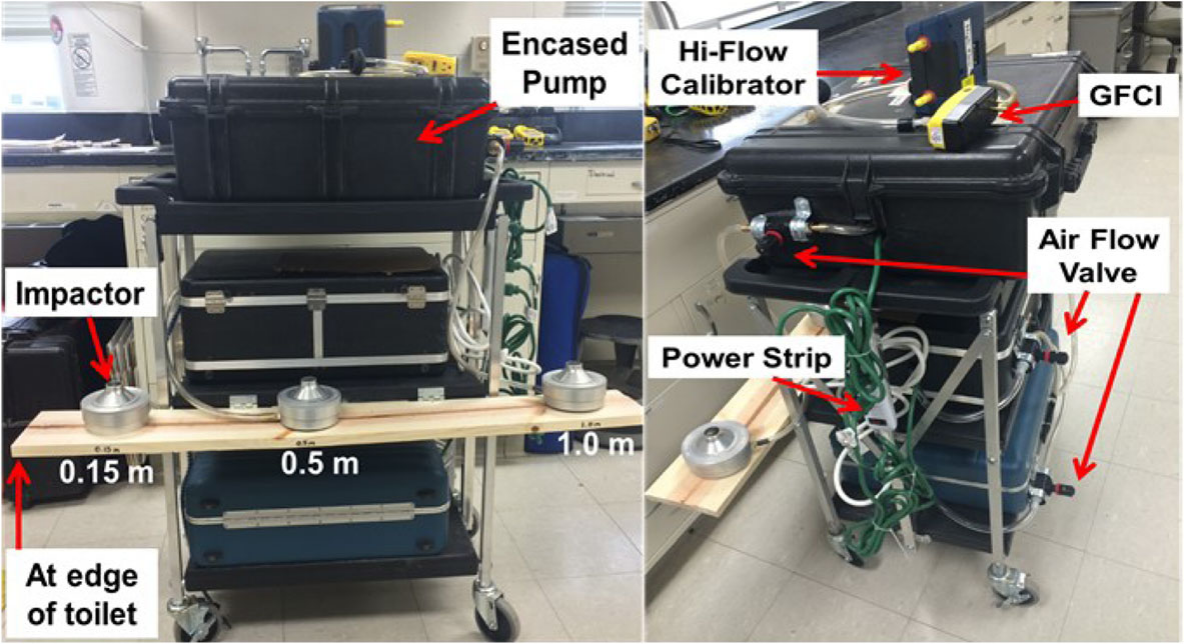
Sampling cart used to measure bioaerosol concentrations in hospital bathrooms

A mobile sampling cart was designed and deployed in a healthcare setting to sample bioaerosols (Fig. 1). The sampling cart consisted of three bioaerosol impactor samplers (SKC BioStage; SKC Inc. Eighty Four, PA). The samplers were attached to the side of the cart 0.6 m (m) from the floor and 0.23 m above the toilet. The cart was maneuvered in front of the toilet to position the samplers 0.15, 0.5, and 1.0 m, respectively, from inside the front edge of the toilet bowl. Samplers were operated with a flow control valve (0.95 cm two-way value; ARP Ingersoll Rand; USA; M/N: 104,104-N03) and air pumps (VP0935A-V1028-D2–0511; Medo Inc.; USA; S/N: 00601036, AC0401A-A1110-E1–178; Medo Inc.; USA; S/N: I1001004T, I1001005T) inside customized air sampling cases designed for quiet operation to ensure patient comfort.

Bathroom Sampling.

To investigate particle and bioaerosols produced from flushing loose fecal wastes, researchers were notified if a patient experienced a bowel movement in the toilet. If the patients were non-ambulatory or used an ostomy bag, the fecal wastes were placed in the toilet. The room number and condition were recorded for each sampling trial. Air sampling was repeated in the same room during the experimental conditions of no waste and flush and no waste with flush. Study procedures were discussed with hospital staff to ensure completion of the study objectives.

Data collection occurred as follows: the sampling cart was maneuvered into the bathroom and placed at the front of the toilet. An optical particle counter (AeroTrak Particle Counter 9306-V; TSI Inc.; USA) was also placed near the toilet (i.e., 0.18 m from the edge of the toilet and 0.30 m from the floor) to measure particle concentration [particles per cubic meter (m^3^) of air]. Particle measurements were collected every minute in six particle size bins (0.3, 0.5, 1, 3, 5, 10 μm). Bioaerosols were measured using SKC BioStage impactors (SKC Inc., Eighty Four, PA) and tryptic soy agar (TSA) plates (Difco Tryptic Soy Agar; Becton, Dickinson and Company; USA; L/N 5234170) at 28.3 L/min. Temperature and relative humidity of the bathroom were measured with a sling psychrometer (Bacharach Inc.; PGH; Pennsylvania; USA) and the dimensions of the bathroom were recorded.

Each trial began with the activation of the particle counter to record background particle concentrations for 3 min. After 3 min, the toilet either remained idle (i.e.*,* not flushed) or was flushed (depending on the experimental condition) and the bioaerosol samplers were activated. After 5 min of air sampling, the sampling instruments were deactivated. The agar plates were replaced with sterile TSA plates and the air sampling instruments were reactivated for an additional 10 min. The agar plates were replaced again and the air sampling instruments were reactivated for an additional 15 min for a total of 9 sampling plates for each sampling trial. All bioaerosol samplers were pre and post-calibrated.

### Analysis of colonies sampled from bathroom air

All plates were incubated (Heratherm IGS180; Thermo Scientific; Germany; S/N: 41,616,442) for approximately 24 h at 37 °C. *Staphylococcus epidermis* (ATCC; USA; L/N: 63,229,747) was quadrant streaked using sterile L-bacterial spreaders (Celltreat Scientific; China; L/N: 150,428–261) on a TSA plate to serve as a positive control. A TSA plate served as a field blank and was placed on the cart during sampling for a negative control. The colony forming units (CFU) that formed on the plates were positive-hole corrected to calculate the bioaerosol concentrations and expressed as CFU/m^3^ [30, 31]. Arithmetic mean bioaerosol concentrations were calculated across time and distance for each sampling event.

### Statistical analysis

The distribution of measured particle and bioaerosol concentrations to be used for statistical analysis were tested for normality using probability plots using a statistical package (Minitab 17 Statistical Software; Minitab Inc.; USA). Paired t-tests were performed to compare particle concentrations measured prior to the flush (i.e.*,* minutes 1, 2, and 3) to the particle concentration measured immediately after the toilet flush *(*i.e.*,* minute 4) for each experimental trial. Paired t-tests were repeated within each experimental condition for particle concentrations recorded by the particle counter at bin sizes of 0.3, 0.5, 1, 3, 5, and 10 μm. A one-way analysis of variance was used to determine statistical differences among bioaerosol concentrations measured across the three experimental conditions of no waste no flush, no waste with flush, and fecal waste with flush. Post-hoc analysis using a Tukey pairwise test identified the conditions in which the bioaerosol concentrations were different within the study. A two-way analysis of variance was used to identify significant differences across conditions with respect to time (5, 10 and 15 min), and distance (0.15, 0.5 and 1.0 m) within the three experimental conditions of the study combined. Observed differences were considered statistically significant when *p*-values were less than 0.05.

## Results

For the 30 sampling trials conducted, 12 bathrooms in the hospital unit were sampled. The bathroom volumes sampled in this study ranged from 8.59 to 12.83 m^3^ with an average volume of 10.55 m^3^ and a standard deviation (SD) of 1.45. The relative humidity of the bathrooms ranged from 62.5 to 88% with an average relative humidity of 72.9% (SD ± 5.84).

**Fig. 2:**
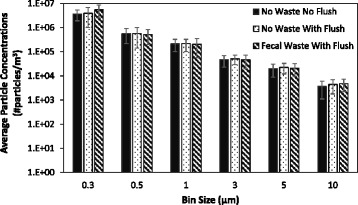
Average particle concentrations across all experimental trials of three experimental bathroom conditions plotted on a log scale. Error bars represent the standard deviation

**Fig. 3:**
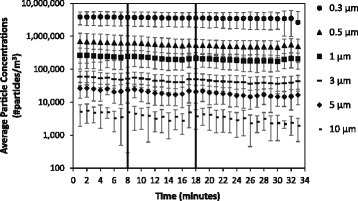
Average particle concentrations plotted on a log scale among 10 sampling trials performed in no waste no flush bathroom conditions. Error bars represent the standard deviation

**Fig. 4:**
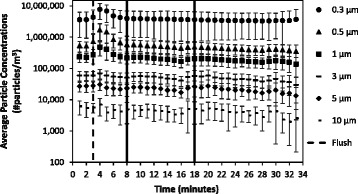
Average particle concentrations plotted on a log scale among 10 sampling trials performed in no waste with flush bathroom conditions. Error bars represent the standard deviation

**Fig. 5:**
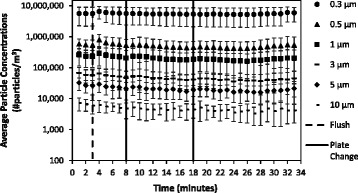
Average particle concentrations plotted on a log scale among 10 sampling trials performed in fecal waste with flush bathroom conditions. Error bars represent the standard deviation

Among the particle concentrations measured, particles < 3 μm in diameter dominated the particle size distribution across the experimental conditions (Fig. 2). Average particle concentrations remained constant when toilets were not flushed (Fig. 3). Among trials where toilets were flushed, particle concentrations increased immediately after the flush (i.e.*,* minute 4) (Figs. 4 and 5). Particle concentrations significantly increased from background concentrations (i.e.*,* minute 1, 2, 3) to after the flush (i.e.*,* minute 4) among particle sizes 0.3, 0.5, 1 and 3 μm among the no waste with flush and waste with flush experimental conditions. Specifically, among the no waste with flush experimental trials particle concentrations increased among 0.3 (*p*-values = 0.002, 0.002, 0.015), 0.5 (*p*-values = 0.002, 0.002, 0.018), 1 (*p*-values = 0.003, 0.003, 0.027) and 3 μm (*p*-values = 0.016, 0.032) particle size bins after the flush. Among the waste with flush trials, significant differences were found in particle concentrations in the 0.3 (*p*-values = 0.009, 0.007, 0.007), 0.5 (*p*-values = 0.018, 0.006, 0.004) and 1 μm (*p*-values = 0.023, 0.013) bins.

The arithmetic mean bioaerosol concentration measured during the no waste no flush toilet condition was 210 CFU/m^3^ (SD ± 136) (Table 1). The mean bioaerosol concentration for the no waste with flush and fecal waste with flush toilet conditions were 240 CFU/m^3^ (SD ± 132) and 278 CFU/m^3^ (SD ± 149), respectively (Table 1). The post-hoc analyses identified the no waste no flush and the fecal waste with flush bioaerosol concentrations to be different (*p*-value = 0.005), however, no statistically significant interactions were identified between the experimental conditions, time sampled (*p*-value = 0.764) and sampler distance (*p*-values = 0.925) and no significant effect of sampler distance on bioaerosol concentration (*p*-value = 0.911). Additionally, no significant interaction effect was identified between time and particle concentration (*p*-value = 0.977).

**Table 1:** Arithmetic mean bioaerosol concentrations and standard deviations of colony forming units sampled from hospital bathrooms each condition representing 10 sampling trials

Toilet Conditions	Mean Bioaerosol Conc. (St. Dev.), CFU/m^3^)
No Waste No Flush	210 (136)^a^
No Waste With Flush	240 (132)
Fecal Waste With Flush	278 (149)^a^

^a^ Room conditions identified by pairwise Tukey test as significantly different (p-value = 0.005)

## Discussion

Flushing significantly increased the number of particles 3 μm and less in diameter and bioaerosol concentrations. However, we did not observe a statistically significant difference in bioaerosol concentrations across time (i.e., 30 min sampling period) or distance (i.e., .15, .5 and 1.0 m from the toilet). The results of our study are significant, as no previous study has measured bioaerosol concentrations in normal hospital bathrooms without flushing, or bioaerosols generated from flushing unmanipulated loose human waste in a hospital setting during active patient care.

Johnson et al., [25] reported similar findings and stated their study found the majority of particles produced from toilet flushes were less than 2 μm. Unlike Johnson et al., [25] the composition of the particle concentrations measured in our study are unknown. Particles sampled may result from water droplets or microorganisms produced from the agitation of toilet water resulting from the flush.

Airborne particle concentrations remained constant in the bathroom when the toilet was not flushed (Fig. 3). Toilet flushing without waste had a greater particle concentration increase than flushing with waste. The presence of toilet paper, consistency of waste, and amount of loose fecal waste may lessen the movement of toilet water during the flush preventing the generation of particles. While particles can be generated from a multitude of sources (e.g.*,* people moving in a room), the increase of particle concentrations immediately following a flushing event provides evidence that flushing (both with and without waste) increases the airborne concentration of particles in hospital bathrooms. Additionally, particles less than 3 μm significantly increased (*p*-values< 0.05). Johnson et al. [25] also found that various toilet designs produce an aerosol where the largest proportion of particles are smaller than 2 μm. Therefore, particles less than 3 μm in diameter may be a significant source of human exposure and environmental contamination as particles of this size can remain airborne for hours [32].

Pure water droplets evaporate in unsaturated conditions where relative humidity is less than 100%, however soluble nuclei (i.e.*,* microorganisms like bacteria) can grow in unsaturated atmospheric conditions by a phenomenon called heterogeneous nucleation [32]. Studies have confirmed that both gram positive and gram negative bacteria may act as a source of soluble nuclei, which have been found to grow to a diameter of 20 μm [33, 34]. These large particles have a much greater settling velocity in comparison to small particles [32]. Faster settling rates may prevent large particle concentrations (i.e.*,* 20 μm or greater) from being observed. Johnson et al., [25] reported particles larger than 5 μm to reach max concentration within 30 s after a flush. Our room conditions where toilets were flushed had observable changes in particle concentrations immediately following the flush only in bin sizes 3, 1, 0.5, and 0.3 μm (Figs. 4 and 5). The faster settling rates of particles within the 5 and 10 μm bins may have caused us to not detect differences within comparisons of the particle concentration of those size bins. Furthermore this phenomenon may contribute to surface contamination near the toilet (i.e., the floor).

Bioaerosols generated from toilets containing fecal waste resulted in the highest average concentration of 278 CFU/m^3^ (SD ± 149), compared to the average concentration of 210 CFU /m^3^ (SD ± 138) near toilets that were idle with no waste present. The no waste no flush and the fecal waste with flush conditions were significantly different from one another (*p*-value = 0.005). The results of our study are significant, as no previous study has measured bioaerosol concentrations in normal hospital bathrooms without flushing, or bioaerosols generated from flushing unmanipulated loose human waste in a hospital setting during active patient care. While differences in bioaerosol concentrations across our experimental condition do not differ substantially, the amount of microorganism required to develop a HAI is often unknown and some healthcare acquired infections (e.g., *Escherichia coli* 0157–10 CFU) occur at low doses [35]. Earlier studies have found that flushing toilets seeded with bacteria, increases the bioaerosol concentration of a bathroom; however these results are not applicable of the bioaerosols produced from flushing manipulated waste [22, 23, 26–29]. The increased concentration of bioaerosols from flushing loose fecal waste supports previous research that identifies a positive correlation between the amount of bacteria used to seed a toilet and measured bioaerosol concentration [22, 23, 26].

Our study detected bioaerosols produced from toilets that were flushed containing no waste, suggesting bacteria remain in the toilet from previous toilet use. We initially hypothesized that differences would be detected between conditions where toilets were flushed containing fecal waste and no waste. However, no differences were observed in the two flushing conditions of waste and no waste, suggesting bacterial residues from previous patient use remained in the water and along the sides of the bowl. When flushed, the agitation of the water could loosen bacteria attached to the wall and be released into the air. Other studies have suggested this phenomenon after seeding toilets with bacteria in the water and along the walls of the toilet [22, 23, 26, 27, 29]. After several flushes, residual bacteria have still been detected in the water or swabbed from toilet surfaces [22, 23, 26, 29].

The study did not observe statistically significant differences in bioaerosol concentration across horizontal distance of the samplers from the toilet or time after the flush (*p*-values = 0.925, 0.977). Prior to this study, there was little information available on the variance in bioaerosol concentrations across horizontal distance and time after a flush. Since our study detected greatest particle concentrations immediately after the flush, the distance and time closest to the toilet flush were hypothesized to have the greatest bioaerosol concentration, which our study results do not support. The plume produced from toilets is unknown due to many toilet varieties and other variables such as toilet height, water usage, and flush energy. The results do suggest that bioaerosol plumes produced during a toilet flush may extend beyond the distances sampled in this study (i.e., 1 m). The magnitude of the bioaerosol plume may be the reason why we did not observed a statistically significant difference across time (i.e., 30 min sampling period) or distance (i.e., .15, .5 and 1.0 m from the toilet). These results are concerning as a large bioaerosol plume may result in the contamination of surfaces bioaerosol exposures for healthcare staff and patients. Toilets with greater energy flushes have been shown to generate higher concentrations of bacteria and particles into the air than toilets with less energy [22, 25, 28]. In hospitals and other public places, toilets with high energy flushes are installed to comply with standards and to reduce the amount of water used and costs [25]. Future research should include longer sampling times at greater distances from the toilet to understand how time or distance from the flush impact bioaerosol concentrations.

During the study, particle and bioaerosol concentrations were collected for 30 sampling events, therefore due to the variability observed in bioaerosol concentrations; our study may have been underpowered which may have resulted in an inability to detect small differences between experimental conditions (i.e.*,* time and distance). The study procedures were conducted in one hospital ward, therefore the observations of this study may not be generalizable to other hospital wards. We chose to perform our study in a single hospital ward with the same toilet design to reduce variability between bathrooms such as make, model and height of toilets. The position of the toilet seat may have affected particle and bioaerosol concentrations. In our study, for the majority of the sampling trials, the toilet seat was left in the “up” position. The bioaerosol concentrations were measured with TSA plates under aerobic conditions. Therefore, our observed concentrations are likely an underestimate due to culture bias. Also, we did not collect data on stool volume added to the toilet prior to flushing. These data may help explain variability in bioaerosol concentrations measured in the field. Our bioaerosol concentration is limited to viable, culturable, fast growing microorganisms (i.e., 24 h) on TSA. Fecal wastes are composed of various species of bacteria with differing oxygen requirements, bioaerosol concentrations being underestimated. In the future studies, samples should be collected with selective growth media or using non-culture and non-target based analysis (e.g., metagenomic shotgun sequencing) to determine the characteristics of bioaerosols generated from toilets [36, 37]. Additionally, this study design allowed a better understanding of how bioaerosol samples could be collected in patient rooms where bathrooms were frequently used by patients and staff. While our design limits the generalizability to other hospitals, our field-based study provides direction to further investigate the role of toilets in aerosolizing fecal waste in a patient-care setting.

## Conclusions

This project is the first field study that has investigated bathroom particle and bioaerosol concentrations from flushing patient fecal wastes in a hospital setting. Flushing significantly increased particles 3 μm and less. Bioaerosol concentrations were significantly higher in bathrooms when toilets were flushed, however we found no difference in bioaerosol concentration across time and distance. This study supports the hypothesis that bioaerosols are generated from flushing toilets and may lead to environmental contamination and inhalation exposures among patients and health care workers.

## Abbreviations

CFU: Colony forming unit
HAI: Health care acquired infection
SD: Standard deviation
US: United States
